# Application of Optical Methods for Determination of Concentration of Doxorubicin in Blood and Plasma

**DOI:** 10.3390/ph15020112

**Published:** 2022-01-18

**Authors:** Tomasz Sikora, Karolina Morawska, Wiesław Lisowski, Paweł Rytel, Agnieszka Dylong

**Affiliations:** 1 Military Institute of Chemistry and Radiometry, 00-910 Warsaw, Poland; k.morawska@wichir.waw.pl (K.M.); w.lisowski@wichir.waw.pl (W.L.); p.rytel@wichir.waw.pl (P.R.); 2 Military Institute of Engineer Technology Named after Professor Józef Kosacki, 50-961 Wroclaw, Poland; dylong@witi.wroc.pl

**Keywords:** anticancer drugs, doxorubicin, UV-Vis spectroscopy, drug monitoring, doxorubicin quantification

## Abstract

The aim of presented research is to develop a simple and quick method of spectrophotometric detection for the determination of doxorubicin hydrochloride in blood and plasma. Anthracycline antibiotics are among the most effective antineoplastic agents. However, despite their high efficacy in the treatment of various types of cancer, their administration is limited primarily because they exhibit myocardial toxicity. This may be a limiting factor in the dosage of medications; nevertheless, drugs exhibiting this mechanism of action constitute a very important group of chemotherapeutics. One of the more widely studied antibiotics from the anthracycline group is doxorubicin. It exhibits the highest antineoplastic activity from among a number of derivative compounds. Because of the adverse effects of doxorubicin, especially cardiotoxicity, it is important to maintain control of its concentration in body fluids. The method in the study consists of extraction doxorubicin from the plasma or blood and measurements of the absorbance of light in the visible light range in a DOX solution with respect to a reference sample. The research used blood and plasma samples spiked with doxorubicin to give concentrations in the range of 0.2–10 µg/mL. Obtained LODs were 1.6 µg/mL and 1.2 µg/mL, respectively.

## 1. Introduction

**Figure 1:**
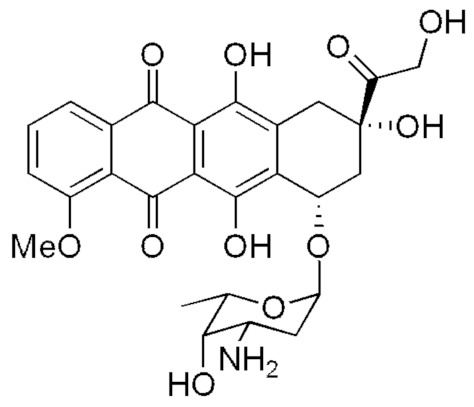
Chemical structure of doxorubicin.

Doxorubicin (DOX) is a cytostatic drug from the group of anthracycline antibiotics, an intercalator with a planar structure containing naphthacenoquinone linked to daunosamine (an amino sugar) via a glycosidic bond (Figure 1). The presence of the aliphatic side chain plays an important role in binding to the DNA helix. Like all intercalators, doxorubicin is a polar molecule with high charge delocalization and a good electron acceptor [1].

Although contemporary studies have established doxorubicin as a first-line antineoplastic agent in chemotherapy, its use has major drawbacks consisting of destroying both cancerous and normal cells and being associated with secondary reactions including cardiotoxicity, cytotoxicity and neurotoxicity, and nephrotoxicity and ototoxicity [2]. DOX has high activity against many types of cancer [3], and is used in the treatment of breast cancer, sarcomas, leukemias, lymphomas and many others [4], usually with other anticancer drugs appropriate to the tumor type.

Clinical use of doxorubicin is limited by a number of side effects, including decreased blood cell production (myelosuppression), nausea, vomiting, extravasation and hair loss [5]. The most significant adverse effect limiting the use of doxorubicin is toxicity and dose-dependent cardiotoxicity, leading to irreversible cardiomyopathy [6,7]. The risk of myocardial damage increases rapidly once the cumulative dose is exceeded, which so far has been adopted as 550 mg/m^2^ [8] which corresponds to a concentration of approximately 1 µg/mL in the peripheral blood. Cardiac dysfunction may manifest during the last phases of drug therapy or may be latent and not develop for several months up to 20 years after discontinuation of chemotherapy [9].

Doxorubicin can break Fe-S bonds in the active centers of IRP (iron regulatory protein), which is an aconitase-like enzyme that regulates iron levels in the cell. The breaking of the bonds is a signal for the accumulation of iron in the cell which leads to an overload of iron in the cell [10,11,12].

Several methods have been reported for quantitative determination of doxorubicin in body fluids and tissues, e.g., by capillary electrophoresis with UV absorption detection [13], with laser-induced fluorescence detection in serum [14] and rabbit plasma [15] voltammetry for urine [16,17,18], high-performance liquid chromatography with a fluorescence spectrophotometric detector at an excitation wavelength of 470 nm and an emission wavelength of 585 nm for lymph and gall [19], with fluorescence detection for rat plasma [20] and serum with fluorescence detection with excitation and emission wavelength set at 480 and 550 nm [21], human plasma [22], methods based on liquid chromatography with fluorescence detection [23], liquid chromatography-tandem mass spectrometry [24], HPLC with fluorescence detection in tumors and tissues [25], and spectrofluorimetry for rabbit serum [26] or rat whole blood [27]. Its high efficacy in oncological treatment has warranted its continued use so far, which is why continuous monitoring of doxorubicin concentrations in body fluids is so important. Studies show that cases of heart failure were 3%, 7% and 18% in patients who had received a cumulative dose of 400, 550 or 700 mg/m^2^ of doxorubicin, respectively. Therefore, oncologists usually limit the cumulative dose of anthracyclines to 550 mg/m^2^ [28]. The introduction of cardiac imaging technology allowing detection of heart failure, or even asymptomatic left ventricular (LV) dysfunction, led to the realization that the incidence of anthracycline-induced cardiotoxicity was higher than previously estimated. In 2003, the incidence of heart failure was estimated at 5%, 16% and 26% for cumulative doses of doxorubicin of 400, 500 and 550 mg/m^2^, respectively [29]. The above data indicate how important it becomes to accurately determine the DOX levels in blood in patients undergoing chemotherapy. In our study, an attempt was made to extract and determine doxorubicin in blood and plasma. For optical determination, a method based on direct measurement of absorbance using quantum yield-enhancing compounds was chosen [30]. The value of quantum yield, apart from the type of substance, is affected by the environment of the fluorophore (type of solvent, temperature, concentration, type and concentration of foreign admixtures and pH of the environment) [31].

The optical method in the study consists of the absorbance of light in the range of 350–700 nm in DOX solution in relation to a reference sample [32]. Characteristic peaks are selected from the absorbance spectrum, for which the wavelength at which the absorption maximum occurs is determined. Analyte concentrations are determined by comparison with the calibration curve.

## 2. Results and Discussion

**Figure 2:**
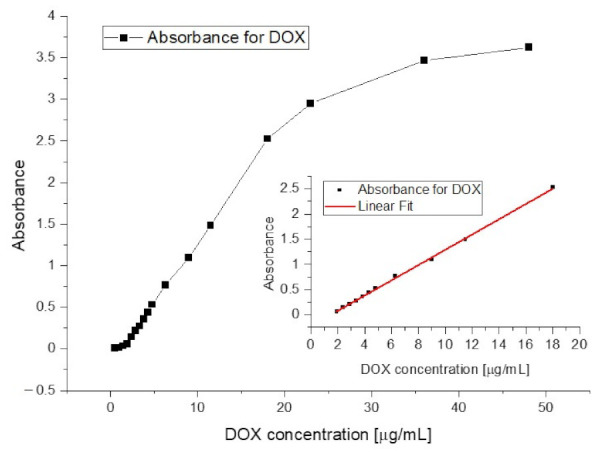
Calibration curve for doxorubicin aqueous solution recorded at a wavelength of 496 nm with extracted linear range.

The absorption spectrum of doxorubicin in aqueous solution gives a single, broad peak with absorption maximum at a wavelength of 496 nm. Its low resolution is most likely due to the high polarity of the molecule and thus a large solvation sphere. For this reason, the image obtained does not allow for a more accurate analysis of the information. At concentrations below 2 µg/mL, the graph (Figure 2) loses its linear character, which makes it impossible to use this curve for analytical purposes. The range of linearity of the readings is too short to be used in further studies.

When ethyl acetate was used as a quantum yield enhancer, a significant improvement in signal resolution was achieved. Transfer to the organic phase allowed a linear calibration curve to be obtained for concentrations up to a LOD of 0.5 µg/mL. 

**Figure 3:**
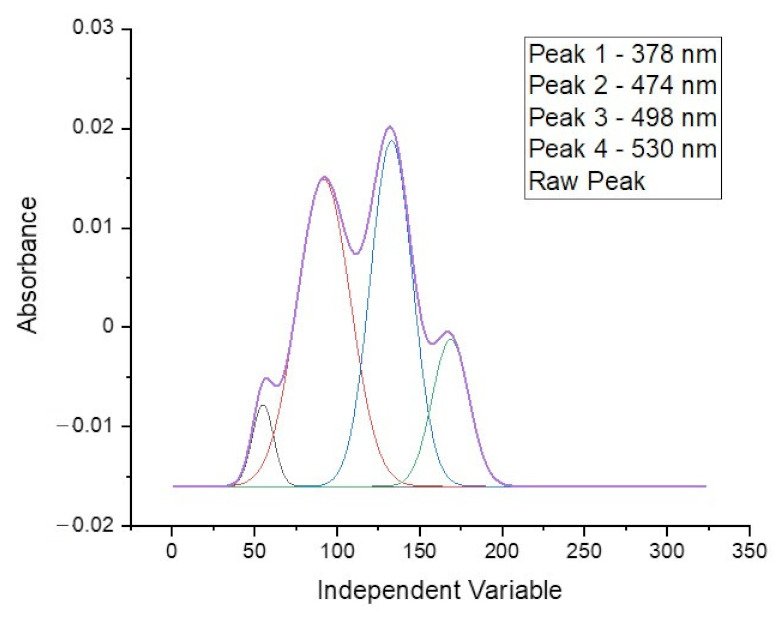
The linear range of the doxorubicin calibration curve recorded at a wavelength of 496 nm for an aqueous solution.

**Figure 4:**
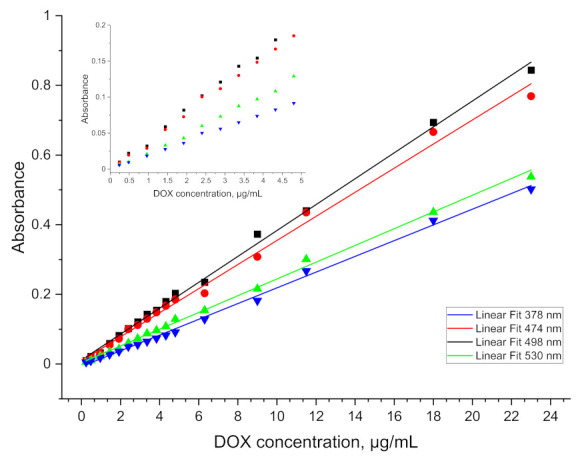
Calibration curve obtained for doxorubicin solution.

The spectrum obtained for doxorubicin in ethyl acetate solution shows four well separated peaks at wavelengths of: 378, 474, 498 and 530 nm, as is presented in Figure 3. The spectra obtained directly from the spectrophotometer were mathematically processed in order to decompose the raw material and isolate only the analyzed ranges. The partition coefficient is strongly shifted towards the aqueous phase so that the absolute absorbance for the organic phase is more than five times lower. This disadvantage is compensated by the elimination of the solvation effect and the separation of as many as four characteristic peaks, as well as by the reduction of the detection threshold to below 0.5 µg/mL. The linear range of the calibration curve obtained for the doxorubicin solution is shown in the Figure 4. This limitation is mainly due to the indication capabilities of the spectrometer, whose resolution did not allow to continue measurements at lower concentrations. 

Due to the low solubility of DOX in organic solvent, it was necessary to carry out studies to optimize the extraction process in the entire range of tested concentrations. The results of the effect of time on the transfer of doxorubicin to ethyl acetate are shown in Table 1.

As shown by the results summarized in Table 1 for this scheme, after 30 min of shaking a complete separation between the aqueous and organic phases is achieved. The average values obtained in all measurements after reaching equilibrium have small standard deviations: A_378_ = 0.034 ± 0.001 A_474_ = 0.074 ± 0.001, A_498_ = 0.083 ± 0.001, A_530_ = 0.051 ± 0.001. In addition, they show good coincidence with the theoretical values calculated from the calibration curve (Table 2).

For further tests, extraction will be carried out using a shaking time of t = 30 min for all concentrations. It is sufficient to transfer enough DOX to the organic phase to provide a LOD of 0.5 µg/mL and short enough to be considered for rapid analysis of doxorubicin in blood or plasma.

The extraction optimization process also included the volume of extractant used. Tests were conducted on 5 mL samples of aqueous doxorubicin solution at different concentrations using multiple extraction, as the partition coefficient is much more favorable for the aqueous phase. 

Optimization of the extraction process again focused on determining the shortest possible sample preparation time, sufficient to determine the lowest concentration of doxorubicin. When using increasing cumulative volumes of ethyl acetate, the increase in absorbance obtained for the combined phases only occurs until 6 mL of organic phase is used, and a decrease is observed for higher volumes. 

This is a surprising effect, as the absorbance values and the relatively high concentration of the stock DOX solution suggest that the extraction should still be efficient. 

Prior to the tests with plasma and blood, doxorubicin determination in protein solutions was performed in order to verify the correctness of assumptions. 

When conducting doxorubicin determinations in a protein solution, a single aliquot of ethyl acetate equal to the volume of the test sample (5 mL), shaken for 30 min, was used for extraction. In the next step, the sample was placed in a centrifuge to separate the denatured protein. The DOX concentrations were chosen to correspond to those used in the determination of the calibration curve. 

**Figure 5:**
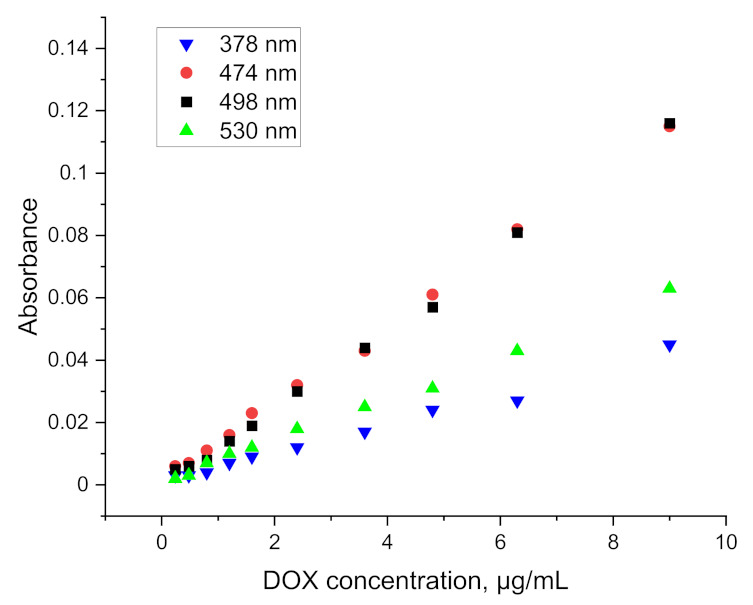
Absorbance curve obtained for DOX solutions after extraction from aqueous protein solution.

As shown in Figure 5, the use of ethyl acetate allows not only for an increased resolution of the absorption spectrum, but also an efficient extraction of doxorubicin combined in the protein complex. The values obtained are slightly lower than those used in the preparation of the calibration curve, nevertheless, they allow for determinations to be carried out at a concentration level of 1 µg/mL. Results for lower concentrations have been obtained, but they are erroneous to a large extent. The relative standard deviation for each point on the graph above 1 µg/mL (at least five measurements for each concentration) falls within the range 4.06–8.48%. 

**Figure 6:**
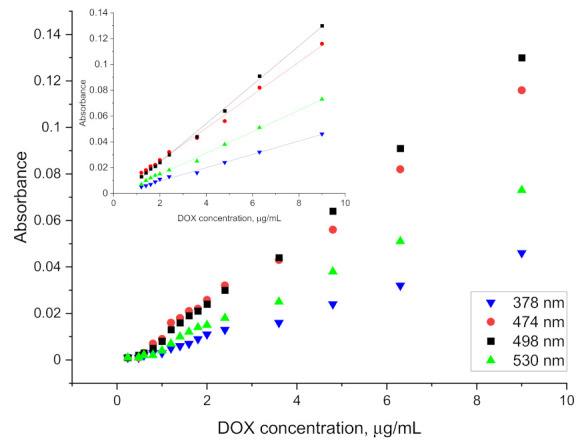
Absorbance curve obtained for DOX solutions after extraction from plasma.

**Figure 7:**
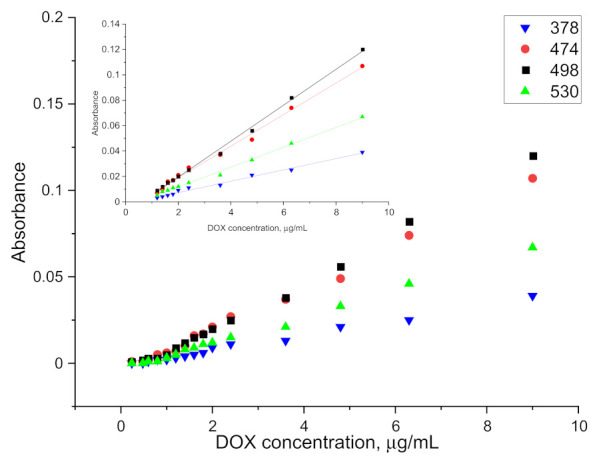
Absorbance curve obtained for DOX solutions after extraction from blood.

A 500 µg/mL aqueous solution of doxorubicin was added to 5 mL blood/plasma samples to give a final concentration range of 0.2 to 10 µg/mL. The parameters presented above were used to perform the extraction. Then 5 mL of ethyl acetate was added. The samples were mixed on a rotary mixer for 30 min and then spun on a centrifuge. There was complete separation of the plasma solids (fat, protein, etc.) from the rest of the solution. The organic phase was separated and transferred to a cuvette after drying. The spectrophotometric measurement of the organic phase after extraction was performed against ethyl acetate and against plasma after extraction, without doxorubicin in the given DOX concentration range. The absorbance values obtained for the samples after the extraction of doxorubicin from blood and plasma and the linear ranges of indications are presented in Figure 6 and Figure 7.

From the obtained results, the LOD for plasma and blood was determined at the level of 1.2 µg/mL and 1.6 µg/mL, respectively. The higher LOD levels as compared to the aqueous phase or protein solution extraction may be caused by the existence of other interactions between doxorubicin and blood/plasma components. The relative standard deviation for measurement after extraction from plasma for doxorubicin concentration ranging from 1.2 µg/mL to 3.6 µg/mL (at least five measurements for each concentration) was around 17.48%. For higher concentrations it was around 7.35%. Results obtained for measurement after extraction from blood were similar and equal to 15.56% for concentration ranging from 1.6 µg/mL to 4.8 µg/mL and 8.07% for higher concentrations. The values of the slopes and intercepts for the calibration curve linear range of indications with standard deviations are presented in Table 3. 

The doxorubicin extraction from blood and plasma using an organic solvent brought very good results. Ethyl acetate significantly increased the quantum yield, making it possible to obtain spectra with clearly separated peaks which allowed for obtaining a low limit of detection. After the mathematical analysis, the peaks can be analyzed separately or as a dataset. The extraction process is relatively short, and the spectrophotometric measurement itself takes up to several dozen seconds. The use of this method can be used to build a measuring device that could automatically carry out all the necessary processes, which would additionally speed up the control of doxorubicin concentration in the blood. The detection limits for the presented method are below the concentration limit, which may cause adverse effects, but further work may move this limit even lower.

## 3. Materials and Methods

Ethyl acetate, distilled water Milli-Q, doxorubicin chloride salt, albumin, monosodium phosphate, dipotassium phosphate and magnesium sulphate anhydrous were purchased from Sigma-Aldrich (Poznań, Poland). Aqueous solution of albumin at a concentration of 30 mg/mL was prepared immediately prior to its use. Phosphate buffer was created with pH 7.4 at a concentration of 0.05 mol/L. All aqueous solutions and their dilutions were made in PBS. Blood and plasma samples were provided by the University of Lodz.

Spectrophotometer Cintra 40—GBC Scientific Equipment Ltd., Dandenong/Australia for visible light absorption measurements was equipped with cuvettes with an absorbent layer 10 mm thick, shaker (OS5-CONTROL) from IKA GmbH&Co, Staufen, Germany, rotary stirrer, glass and automatic pipettes (FINNPIPETTE F2) Thermo Fisher, Waltham, MA, USA, pH meter Hanna Instruments Ltd,, Smithfield, RI and centrifuge Hettich EBA 20, Andreas Hettich GmbH & Co.; Tuttlingen, Germany.

Standard solutions were prepared from dry powder of doxorubicin hydrochloride. A test portion weighed at an accuracy of 4 decimal places was quantitatively transferred into a 100 mL volumetric flask. A solution with a concentration of 48 µg/mL was obtained, which formed the basis for subsequent dilutions in the range of 0.2–48 µg/mL. For each dilution the absorbance was measured at least five times, each time reading the result at a wavelength of 496 nm. 

Subsequently, DOX extraction trials were carried out from aqueous solution using ethyl acetate. Both time and volume of extractant were taken into account to optimize the extraction process. For this purpose, four samples of a 5 mL aqueous DOX solution were prepared, then 5 mL of ethyl acetate was added and placed on a shaker set at 430 rpm. Subsequent samples were shaken for 15, 30, 45 and 60 min. The organic phase was dried with magnesium sulphate. The clear fraction of the organic phase was transferred to a measuring cuvette and the absorbance was measured. 

Once the optimum extraction time had been determined, tests were carried out on the effect of extractant volumes ranging from 2 mL to 10 mL for 5 mL of doxorubicin solution. Extraction was carried out by shaking for 30 min. The organic phase was then separated in a separatory funnel and dried from residual water as described above. Absorbance measurement was performed for the dried sample. 

**Figure 8:**
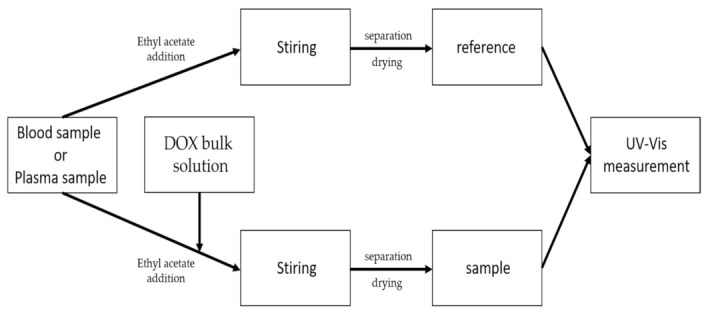
Scheme for the detection of doxorubicin in blood/plasma.

Following the optimization of the extraction parameters from the aqueous solution, the tests with plasma and blood were started. In order to obtain the appropriate concentration, a doxorubicin solution was prepared and then added to plasma and blood samples as described below and following the scheme shown in Figure 8.

All samples were then mixed and placed in a refrigerator for four hours. Extraction was carried out under optimized conditions derived from previous tests, i.e., time and volume of extractant. All samples were brought to room temperature before conducting absorbance measurements. 

For extraction, an aliquot of 500 µg/mL doxorubicin solution was added to 5 mL of plasma to give concentrations in the range of 0.2–10 µg/mL. Extraction with ethyl acetate was then carried out. The samples were mixed on a rotary stirrer for 30 min and then centrifuged on a centrifuge (5000 rpm, 2 min). There was a complete separation of the solid components of the plasma from the rest of the solution. The collected organic phase was subjected to drying with magnesium sulphate followed by spectrophotometric measurement against the reference sample. The organic phase (of ethyl acetate) after extraction from plasma without the addition of doxorubicin was used as a reference sample. Blood samples were prepared in the same way as plasma samples.

## 4. Conclusions

The presented results show that this method has the potential to determine doxorubicin even in complex matrices.

The methods described in the introduction allow for achieving much lower detection limits, ranging from 0.5 ng/mL for LC-FL and LC-MS methods to about 10 ng/mL for electrochemical methods. These results are much lower than those obtained in the presented method. Currently, the limit of detection (LOD) for the described method has been determined to be 0.5 µg/mL versus aqueous solution for pure substances, and approximately 0.8 µg/mL versus aqueous solution for protein solution determinations. For the measurements performed on plasma and blood samples, LOD was obtained at 1.2 µg/mL and 1.6 µg/mL, respectively. These results indicate that the method can be used to control the concentration of doxorubicin in the peripheral blood collected from a patient undergoing chemotherapy, however the current level of research advancement does not yet allow for practical application. Nevertheless, there are several elements to note. The above methods require time-consuming sample preparation and complicated and/or expensive equipment. The main novelty in the method presented in this article is that blood taken directly from the patient undergoes a one-step preparation process lasting up to 40 min and the measurement itself takes less than two minutes. Importantly, the only consumable material in this method is ethyl acetate (+ spectrophotometric cuvettes), which translates directly into the low cost of the entire process. This is a significant advantage of this method over the methods described earlier—the time needed for the analysis from blood collection to obtaining the result. The use of ethyl acetate for the extraction of doxorubicin not only increased the resolution of the peaks obtained on the spectrophotometer, but also allowed for direct extraction from blood and plasma—matrix elements mostly remain in the aqueous phase after extraction. The aforementioned increase of the peak resolution allows the use of an optical signal amplifier in the development versions of the presented method. Currently, work is focused on lowering the LOD and LOQ. Preliminary research has been carried out with the use of a new spectrophotometer, and the obtained results indicate that the use of a more sensitive optical system allows for a lower LOD, at least an order of magnitude.

The presented method is quick, and the sample preparation is simple and does not require the use of complicated techniques. This allows for precisely selecting the amount of the drug to be administered and reduces the risk of side effects associated with the high concentration of doxorubicin.

## Figures and Tables

**Table 1:** Absorbance values obtained for ethyl acetate samples after extraction from the 2 µg/mL aqueous phase after a specified period of time.

Extraction Time (Minutes)	Wavelength (nm)			
	378	474	498	530
15	0.018	0.041	0.066	0.053
	0.011	0.043	0.068	0.056
	0.019	0.042	0.067	0.055
	0.020	0.041	0.068	0.055
30	0.032	0.073	0.081	0.050
	0.037	0.074	0.082	0.050
	0.034	0.075	0.082	0.051
	0.034	0.074	0.084	0.050
45	0.036	0.075	0.083	0.051
	0.038	0.077	0.083	0.052
	0.034	0.074	0.082	0.051
	0.035	0.075	0.082	0.050
60	0.036	0.076	0.083	0.053
	0.038	0.075	0.086	0.052
	0.037	0.078	0.086	0.052

**Table 2:** Comparison of the absorbance values obtained, and the values calculated from the calibration curve.

Wavelength (nm)	Average Absorbance Value Obtained During the Tests	Absorbance Value Determined from the Calibration Curve
378	0.034	0.036
474	0.074	0.075
498	0.083	0.082
530	0.051	0.049

**Table 3:** Equation parameters for the linear range of calibration curves.

	Wavelength	Slope ± SD	Intercept ± SD
Plasma	378	0.0048 ± 0.0001	0.0003 ± 0.0001
	474	0.0126 ± 0.0005	0.0011 ± 0.0001
	498	0.0137 ± 0.0005	−0.0027 ± 0.0004
	530	0.0068 ± 0.0002	0.0008 ± 0.0001
Blood	378	0.0045 ± 0.0001	−0.0017 ± 0.0002
	474	0.0123 ± 0.0005	−0.0052 ± 0.0006
	498	0.0138 ± 0.005	−0.0071 ± 0.0008
	530	0.0078 ± 0.0002	−0.0039 ± 0.0004

## Data Availability

Data is contained within the article.
